# UFMylation: A Unique & Fashionable Modification for Life

**DOI:** 10.1016/j.gpb.2016.04.001

**Published:** 2016-05-20

**Authors:** Ying Wei, Xingzhi Xu

**Affiliations:** Beijing Key Laboratory of DNA Damage Response and College of Life Sciences, Capital Normal University, Beijing 100048, China

**Keywords:** Ubiquitin-fold modifier 1, Ufmylation, Endoplasmic reticulum stress, Cancer, Post-translation modification, Ubiquitin-like proteins

## Abstract

**Ubiquitin-fold modifier 1** (UFM1) is one of the newly-identified **ubiquitin-like proteins**. Similar to ubiquitin, UFM1 is conjugated to its target proteins by a three-step enzymatic reaction. The UFM1-activating enzyme, ubiquitin-like modifier-activating enzyme 5 (UBA5), serves as the E1 to activate UFM1; UFM1-conjugating enzyme 1 (UFC1) acts as the E2 to transfer the activated UFM1 to the active site of the E2; and the UFM1-specific ligase 1 (UFL1) acts as the E3 to recognize its substrate, transfer, and ligate the UFM1 from E2 to the substrate. This process is called **ufmylation**. UFM1 chains can be cleaved from its target proteins by UFM1-specific proteases (UfSPs), suggesting that the **ufmylation** modification is reversible. UFM1 cascade is conserved among nearly all of the eukaryotic organisms, but not in yeast, and associated with several cellular activities including the **endoplasmic reticulum stress** response and hematopoiesis. Furthermore, the UFM1 cascade is closely related to a series of human diseases. In this review, we summarize the molecular details of this reversible modification process, the recent progress of its functional studies, as well as its implication in tumorigenesis and potential therapeutic targets for **cancer**.

## Introduction

Protein post-translational modifications (PTM) refer to covalent and generally enzymatic modification of proteins during or after protein biosynthesis. Up to now, more than 200 types of PTMs have been reported, including addition of functional groups (*e.g.*, phosphorylation, methylation, and acetylation), conjugation of other proteins or peptides (*e.g.*, ubiquitination), chemical modification of amino acid (AA) residues (*e.g.*, deamidation), and structural changes (*e.g.*, disulfide bridges) [1], [2]. A protein can be modified with one type of PTM or varieties of combinations of PTM types. Moreover, one type of PTM may contain one (*e.g.*, phosphorylation), a few (*e.g.*, methylation), and up to many (*e.g.*, ubiquitination and glycosylation) subtypes. Therefore, PTMs expand exponentially cellular protein varieties and increase a cellular proteome to more than one million different proteins to cope with the complex and fine-tuned cellular activities, such as protein–protein interactions, cell cycle control, cell differentiation, gene transcription, signal transduction, DNA damage repair, apoptosis, endocytosis, and receptor trafficking [3], [4], [5].

Ubiquitination is one of the most common PTMs, next to glycosylation and phosphorylation [6]. Ubiquitin (Ub) is conjugated to its target proteins by an elaborate enzymatic reaction chain, which consists of an activating enzyme E1, a conjugating enzyme E2, and a ligating enzyme E3 [6]. The human genome encodes one E1, dozens of E2, and hundreds of E3. Besides, the Ub chain can be cleaved from its target proteins by deubiquitinating enzymes (DUBs), suggesting that ubiquitination is reversible [7]. A target protein is modified covalently with a single moiety of Ub (monoubiquitination) through its C-terminal glycine (Gly) residue and a lysine (Lys, K) residue within the target protein, or subsequently with multiple moieties of Ub through its C-terminal Gly residue and a Lys residue or the 1st methionine (Met) residue within the pre-added Ub moiety. There are seven Lys residues (K6, K11, K27, K29, K33, K48, and K63) and the initial Met residue in the Ub polypeptide. Thus, there are at least 8 different linkage types for polyubiquitination chains. Yet, a polyubiquitination chain could be a mixture of more than one linkage type, making this modification more complex and divergent. Different linkage types of ubiquitination modification confer a target with different biological functions [8]. Among them, it is well known that the K48-linked poly-ubiquitination modification targets proteins for degradation through the 26S proteasome, while the K63 linkage poly-ubiquitination regulates protein activity and protein–protein interaction networks [9], [10].

A number of small Ub-like proteins (UBLs), such as small Ub-like modifier (SUMO) [11], neural precursor cell-expressed and developmentally-downregulated 8 (NEDD8) [12], and interferon-stimulated gene 15 (ISG15) [13], have been subsequently identified. Although most UBLs have no obvious sequence identity to Ub, they all share a similar tertiary structure. Like Ub, these UBLs can be covalently conjugated to their target proteins through a series of enzymatic reactions analogous to ubiquitylation, endowing them with different biological functions [14]. The UBLs are divided into two subfamilies. Type-1 UBLs, such as SUMO, NEDD8, and UCRP/ISG15, are ligated to target proteins in a process similar to ubiquitylation. Type-2 UBLs, which are also known as Ub domain proteins (UDPs), have a similar structure to Ub and are embedded in different large proteins [13]. These include Rad23, elongin B, and parkin [14].

**Figure 1:**
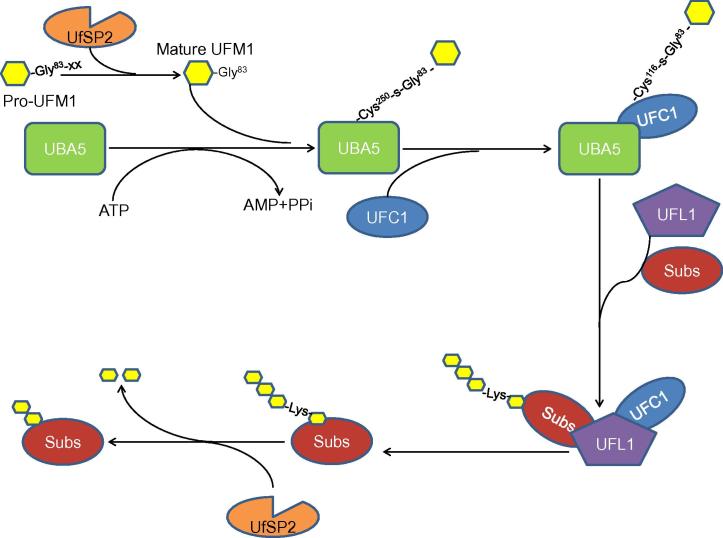
The human ufmylation pathway Pro-UFM1 is cleaved by the UfSPs to expose its C-terminal conserved Gly residue. The mature form of UFM1 is activated by UBA5 at the expense of ATP hydrolysis, forming a high energy thioester bond with Cys 250 of UBA5. The E2 UFC1 then binds to UBA5, and the activated UFM1 is transferred to UFC1, forming a similar thioester linkage with Cys116 in UFC1. Finally, the E3 ligase UFL1 brings in a substrate (Subs) and UFC1-activated UFM1, UFM1 is then conjugated to its substrate. For the reverse process, UfSP2 cleaves UFM1 chains from its substrate. UFM1, ubiquitin-fold modifier 1; Pro-UFM1, UFM1 precursor; UfSP, UFM1-specific protease; UBA5, ubiquitin-like modifier-activating enzyme 5; UFC1, UFM1-conjugating enzyme 1; UFL1, UFM1-specific ligase 1.

Ubiquitin fold modifier 1 (UFM1) is one of the newly-identified type 1 UBLs [15]. Like ubiquitination, UFM1 is conjugated to a target protein by a three-step enzymatic reaction involving E1, the UFM1-activating enzyme (ubiquitin-like modifier-activating enzyme 5; UBA5); E2, the UFM1-conjugating enzyme 1 (UFC1); and E3, the UFM1-specific ligase 1 (UFL1). Usually, UFM1 is present in a precursor form (Pro-UFM1). Cleavage by the UFM1-specific proteases (UfSPs) exposes the conserved Gly residue on its C-terminus and this residue is essential for its subsequent conjugating reactions. Thereafter, with the help of ATP, the mature form of UFM1 is activated by UBA5, forming a high energy thioester bond with the Cystine (Cys) 250 of UBA5. Next, UFC1 interacts with the Ub-fold domain of UBA5, and the activated UFM1 is transferred to the UFC1, forming a similar thioester linkage with Cys116 in UFC1. Finally, with the help of UFL1, UFM1 is conjugated to its target proteins, such as UFM1-binding protein 1 (UfBP1) [16] and activating signal cointegrator 1 (ASC1) [17] (Figure 1). The specific UfSPs can also cleave UFM1 from its target proteins, suggesting that ufmylation is reversible [18], [19] (Figure 1). UFM1 cascade is conserved among nearly all the eukaryotic organisms, except yeast [18], [20], implying the essential role of UFM1 modification in cellular activities. Recent studies have uncovered that UFM1 modification is tightly related to the endoplasmic reticulum (ER) stress [21], [22], hematopoiesis [23], [24], [25], fatty acid metabolism [26], and G-protein coupled receptor (GPCR) biogenesis [27]. Furthermore, the abnormal UFM1 cascade is reported to be associated with a number of human diseases, including cancer, ischemic heart diseases, diabetes, atherosclerosis, hip dysplasia, and schizophrenia [17], [18].

The human genome encodes a single ufmylation cascade (UBA5−UFC1−UFL1); however, its physiological substrates are far from clear. In this review, we will introduce the human ufmylation cascade, its known physiological substrates, and its involvement in a few cellular activities. We will summarize the abnormalities of the ufmylation cascade in different tumors. A perspective of ufmylation in tumorigenesis and cancer therapy will be discussed as well.

## Key players of the ufmylation process

Key players of the ufmylation process include UFM1, UBA5, UFC1, UFL1, and UfSP2 [18].

UFM1, also known as C13orf20, consists of 85 AA residues with a calculated molecular mass of 9.1 kDa. Although human UFM1 only has 16% sequence identity with Ub, the global fold of human UFM1 is similar to that of Ub in their tertiary structures [15]. While Ub and many UBLs have the conserved C-terminal di-Gly (Gly-Gly) residues, the mature UFM1 only has a single Gly residue at its C-terminus followed by a Ser-Cys dipeptide. Present in a precursor form in human cells, UFM1 is processed by the two UfSPs, namely, UfSP1 and UfSP2, to produce mature UFM1 [19]. Immunocytochemical analysis revealed that UFM1 is localized both in the nucleus and the cytoplasm [15]. UFM1 is conserved in metazoan and plants, but not found in yeast, suggesting its specific roles in multicellular organisms.

UBA5, also known as UBE1DC1, is the only E1 enzyme for ufmylation encoded by the human genome [15], [28]. *UBA5* encodes a polypeptide of 404 AA residues with a calculated molecular weight of 44.7 kDa. Typically, a Ub-activating enzyme E1 comprises the first and second catalytic cysteine half-domains (FCCH and SCCH, respectively), adenylation domain, and C-terminal Ub-fold domain [29]. However, UBA5 does not possess all these characteristic domains; instead, it only has an adenylation domain where the catalytic Cys250 is located and a Ub-fold domain [30]. Thus, UBA5 is significantly smaller than other Ub-associated (UBA) family members. UBA5 is mainly detected in the cytoplasm. Interestingly, a typical E1 works as a dimer to activate its specific Ub-like proteins. While the monomeric UBA5 is able to activate UFM1 *in vitro* [15], [30], whether UBA5 works as a dimer or monomer *in vivo* is awaiting further investigation.

A canonical E1 activates a UBL through a three-step mechanism. In the first step, E1 binds ATP and UBL, forming a UBL-adenylate intermediate. Second, the conserved Cys residue in the catalytic cysteine domain reacts with the UBL-adenylate to form an E1-UBL thioester. Third, the E1-UBL thioester catalyzes another round of UBL adenylation with ATP, forming a ternary complex [29]. In contrast, the activation of UFM1 by UBA5 is via a two-step mechanism. First, mature UFM1 forms a noncovalent complex with UBA5. Then, with the help of ATP, UFM1 is conjugated to UBA5 at Cys250 in the catalytic domain via a thioester bond, forming a binary complex [30]. It has been demonstrated that UBA5 is specific for UFM1 activation [23], although there is a report showing that UBA5 activates SUMO2 in the nucleus [31].

Significantly, *Uba5* knockout mice die *in utero* due to severe anemia resulting from defective development in the megakaryocyte and erythroid progenitors [23]. The anemia phenotype of the *Uba5*-deficient mouse embryos can be rescued by transgenic expression of *Uba5* in the erythroid lineage, together with prolonged survival, although *Uba5* is dispensable for the erythropoietin production. These results reveal the important role of the ufmylation process in erythroid differentiation [23].

UFC1, also known as HSPC155, consists of 167 AA residues with a calculated molecular weight of 19.4 kDa. UFC1 is predominantly localized in the nucleus and partially in the cytoplasm [15]. Despite lacking obvious sequence homology with other E2 enzymes, UFC1 does have a relatively conserved catalytic core domain, which is conserved in all E2 enzymes [15]. In the catalytic core domain, there are approximately 10 AA residues surrounding the active site Cys116, forming a flexible loop that is highly solvent-accessible. Once UFC1 binds to the C-terminal domain of UBA5, the activated UFM1 is transferred to the Cys116 of UFC1 by a trans-esterification reaction [18], [20].

The neuronal cell adhesion molecule (NCAM) plays important roles in the neurodevelopment and in synaptic plasticity in the adult brain [32]. NCAM140, an isoform of NCAM, was shown to interact with UFC1 through its cytoplasmic domain and co-localize with UFC1 on the surface of B35 neuroblastoma cells [32]. Besides, overexpression of UFM1 resulted in the increased NCAM140 endocytosis [32]. Therefore, the UFM1 cascade may play an essential role in trafficking of cell surface molecules.

UFL1, also known as Maxer, regulator of C53/LZAP and DDRGK1 (RCAD), novel LZAP-binding protein (NLBP), and KIAA0776, consists of 794 AA residues with a calculated molecular weight of 89.5 kDa. UFL1 is mainly located in the endoplasmic ER [16]. It possesses a transmembrane domain and a nuclear localization signal (NLS), which is functional only when the transmembrane domain is deleted [16]. The N-terminal region of UFL1 is highly conserved across different species and is important for the transfer of UFM1 from UFC1 to its target proteins [16]. Surprisingly, UFL1 does not possess any typical domains conserved in E3 ligases, such as the homologous to the E6AP carboxyl terminus domain (HECT), the really interesting new gene (RING) finger domain, or U-box [15]. For instance, an active cysteine site for transthiolation reaction within N terminus is typically identified in HECT type E3 ligases; however, UFL1 lacks this cysteine site [16]. Thus, it is believed that UFL1 may be a scaffold type E3-like RING finger E3 protein that can recruit E2 enzymes and target proteins.

Recent investigations have demonstrated that *Ufl1* knockout mice die *in utero* due to severe anemia [24]. Knocking down of *Ufl1* resulted in increased ER stress and unfolded protein response (UPR) in bone marrow cells. Furthermore, loss of UFL1 compromised autophagic degradation, while elevating mitochondrial mass and reactive oxygen species, p53 activation, DNA damage response, and death of hematopoietic stem cells (HSCs). Thus, UFL1 is indispensable for embryonic development, erythroid differentiation, and HSC survival [24].

The processing of the C-terminal region of Pro-UFM1, as well as releasing of UFM1 from its target proteins, is mediated by two UfSPs, UfSP1 and UfSP2 [19]. Human UfSP1 consists of 217 AA residues with a calculated molecular weight of 23.4 kDa, while human UfSP2 (C4orf20) consists of 469 AA residues with a calculated molecular weight of 53.2 kDa. UfSP2 differs from UfSP1 due to the presence of an extended N-terminal domain, which has a unique structure and may play a role in recognizing the specific substrate during the deconjugation process [19]. UfSP2 is present in nearly all multicellular organisms, while UfSP1 is not found in plants and nematodes and is usually expressed weaker than UfSP2 [19]. Although UfSP1 and UfSP2 have no obvious sequence homology with the known Ub-like protein specific proteases (ULPs) and DUBs, they exhibit typical features of cysteine proteases, including possession of a conserved catalytic triad Cys-His-Asp and sensitivity of catalytic inhibition by sulfhydryl-blocking agents [19]. Thus, the UfSPs may constitute a new subfamily of the cysteine protease.

UfSP2 may also play a role in the maturation of GPCRs on the ER membrane in a UFM1-independent manner in *Caenorhabditis elegans*. The odorant response abnormal protein (ODR-8; analogous to human UfSP2)-containing complex in the ER promotes GPCR folding, maturation, and export from ER [27]. ODR-8 is reported to have a distinct neuronal expression profile in *C. elegans*, while other UFM1 pathway members are mainly expressed in the intestine [33]. Besides, a mutation of UfSP2 was reported in a case of the Beukes familial hip dysplasia [34], suggesting that the deufmylation process gets involved in human pathogenesis as well.

## Key physiological substrates of ufmylation

The physiological targets of the UFM1 conjugation system remain largely undefined. Only a handful of ufmylation targets have been identified so far, including UFM1-binding protein 1 (UfBP1) and activating signal cointegrator 1 (ASC1).

UfBP1, also known as C20orf116, Dashurin, or DDRGK domain containing 1 (DDRGK1), consists of 314 AA residues with a calculated molecular weight of 35.6 kDa. UfBP1 is the first identified substrate of ufmylation [16]. UfBP1 contains a transmembrane helix (AA residues 4–21), a NLS sequence (AA residues 64–68), a proteasome-COP9-initiation factor (PCI) domain (AA residues 228–272), and a DDRGK domain (AA residues 253–267). The ufmylation of UfBP1 takes place at the K267 in the PCI domain [16]. The PCI domain is best-known for its role in mediating protein–protein interaction and in the formation of several multiprotein complexes, such as the 26S proteasome, the COP9 signalosome (CSN), and the eukaryotic translation initiation factor 3 (eIF3) complex [35]. UfBP1 is predominantly localized in the ER, and its N-terminal signal domain is important for its localization in the cytosolic side of the ER membrane. The ER-localized UfBP1 can recruit UfSP2, and interact with UFL1 and C53 (also known as Cdk5rap3, one of the UFM1 substrates), to form a large multi-protein complex [16]. Defect in the N-terminal signal sequence of UfBP1 results in nuclear localization [16]. Overexpression of *UfBP1* was associated with ER proliferation and neogenesis [36], whereas knockout of *UfBP1* caused an elevated ER stress and activation of UPR [25].

Moreover, UfBP1 can interact with UFM1 and UFL1, as well as the substrates of ufmylation, such as ASC1 and C53, forming a big complex. UfBP1 can promote the ufmylation of ASC1 [17]. Consistent with this phenomenon, knockdown of *UfBP1* abolished the ufmylation of ASC1, and over-expression of the UfBP1(K267R) mutant, which is deficient in the UfBP1 ufmylation, also abolished the ASC1 ufmylation [17]. Germ-line deletion of UfBP1 resulted in defective erythroid development and embryonic lethality, while somatic ablation of UfBP1 impaired adult hematopoiesis, leading to pancytopenia and animal death [25]. Thus, UfBP1 is not only a substrate of ufmylation, but the ufmylated UfBP1 may serve as a cofactor for the ufmylation of other substrates.

ASC1 is a transcriptional coactivator of estrogen receptor-α (ERα) as well as other nuclear receptors (*e.g.*, C53, androgen receptor, and thyroid hormone receptor) [37]. ASC1 possesses a zinc finger (ZF) domain, which is thought to be a binding site for nuclear receptors and transcriptional coactivators such as p300 and steroid receptor coactivator1 (SRC1) [37]. Thus, ASC1 may serve as a platform protein to recruit the necessary components for nuclear receptor-mediated transcription. Besides, ASC1 is thought to be a tumor suppressor, as it can activate p53, induce apoptosis, and suppress NF-κB signaling [17]. Knockdown of ASC1 reduced expression of erythroid transcription factors, but did not elevate basal ER stress [25].

ASC1 is a novel UFM1 target protein [16]. It was recently demonstrated that the polyufmylation of ASC1 promotes breast cancer development [17]. The ASC1 ufmylation requires all factors of the UFM1 cascade, including UFM1, UBA5 (E1), UFC1 (E2), UFL1 (E3), and UfBP1. Absence of any of these factors will abrogate the ASC1 ufmylation. K324, K325, K334, and K367 of ASC1 all serve as the acceptor sites for UFM1 modification, whereas only the K69 of UFM1 is involved in the poly-UFM1 chain formation on ASC1 [17]. In the absence of estrogen, ASC1 in the ERα-positive breast cancer cells undergoes polyufmylation, which is quickly reversed by the bound UfSP2. In the presence of estrogen, the ligand-bound dimeric ERα displaces UfSP2 from binding to ASC1, allowing accumulation of ASC1 ufmylation. Polyufmylated ASC1 may serve as a scaffold protein to recruit p300, SRC1, and ASC1 itself to the promoter of ERα target genes for transcriptional activation, thus leading to excessive cell proliferation and ultimately tumor formation [38].

## Key cellular processes regulated by ufmylation

Based on the physiological targets of ufmylation identified so far, we have gained some insight of ufmylation in ER homeostasis, development and differentiation of blood progenitors. In addition, the ufmylation may be involved in GPCR maturation in *C. elegans* as mentioned above.

ER is an organelle essential for the protein processing, folding, and trafficking, as well as lipid biosynthesis and calcium homeostasis [39], [40]. Thus, ER homeostasis is essential for protein maturation [39]. Disturbance of the ER homeostasis will result in the ER stress response and the activation of UPR [39]. The UfBP1, UfSP2, UFL1, and UFM1 can form a large protein complex at the cytosolic side of the ER membrane, and the UFM1 modification system was reported to play an important role in the ER function [21], [22].

The pathology of both type 2 diabetes and ischemic heart disease is related to the ER stress response [21]. The expression of UFM1 cascade is up-regulated in the pancreatic islets of Langerhans and some other secretory tissues [21]. With the treatment of glycosylation inhibitor tunicamycin (TM), or ER Ca^2+^ ATPase inhibitor thapsigargin (TG) for ER stress induction, increased expression of UFM1, UfBP1, and UFL1 was observed in the mouse INS-1E cells [41]. When vesicle trafficking was inhibited by brefeldin A (BFA, an inhibitor of vesicle trafficking), the transcript levels of UFM1 system increased, which was not observed in mouse embryonic fibroblast cells (MEFs) with knocking down of X-box binding protein 1 (Xbp1), a transcription factor of UPR [22]. The ufmylation-related genes all contain an XBP1-binding element (UPRE) in their promoter regions [22]. Thus, the UFM1 system may participate in vesicle trafficking, loss of which will lead to an increased protein load in the ER and subsequently increase the ER stress [22].

It was reported that loss-of-function of ufmylation resulted in an increased apoptosis of pancreatic beta cells [41]. Knockdown of UFM1 increased the apoptosis of the mouse macrophage cell line RAW264.7 cells [42]. However, in *C. elegans*, the UFM1 conjugation is high when the protein load is low and *vice versa* [41]. Additionally, the UFM1 modification can also participate in other stress response, like oxidative, heat, and pathogen stresses [43]. In *Leishmania donovani*, the UFM1 cascade is located in the mitochondria, and can interact with mitochondrial trifunctional protein (MTP) to affect the beta oxidation of long-chain acyl-CoA esters [26], [44]. Loss of UFM1 results in the decreased amount of acyl-CoA and reduces the survival of amastigotes in human macrophage cells [26], [44]. Thus, UFM1 modification may play a protective role in maintaining the ER homeostasis and avoid the ER stress-induced apoptosis.

Germ-line deletion of *Uba5*, *Ufl1*, or *Ufbp1* in mice all led to embryonic lethality and impaired hematopoietic development (erythroid linage development in particular) [23], [24], [25]. Additionally, *Ufl1* knockout in mice compromised HSC survival, and tamoxifen-induced somatic knockout of *Ufl1* in mice resulted in death due to severe anemia [23], [24], [25]. Both *Ufl1* knockout and *Ufbp1* knockout mice exhibited increased ER stress and activation of UPR [24], [25], further confirming ufmylation in ER homeostasis. It has been proposed that the ufmylation pathway targets ASC1 for ufmylation, stimulates expression of erythroid transcription factors, and thus ensures erythroid development. In addition, the pathway also targets a yet unknown factor for ufmylation, maintaining ER homeostasis, and thus promoting HSC survival [25].

## The ufmylation pathway in human diseases

Abnormalities in the ufmylation pathway have been found to associate with varieties of human diseases, including ischemic heart diseases [21], diabetes [21], atherosclerosis [42], hip dysplasia [34], schizophrenia [45], and cancer [17].

The ufmylation of ASC1 is important for the transactivation of ERα and thus breast cancer development [17]. In addition, UfBP1 knockdown compromises cell proliferation and invasion in human osteosarcoma U2OS cells [46]. However, both UFL1 and UfBP1 have been reported to suppress the NF-κB signaling pathway [46]. Thus, the ufmylation pathway may promote or suppress tumorigenesis through targeting different substrates. Indeed, in The Cancer Genome Atlas (TCGA) cancer database (www.cbioportal.com), for lung squamous cell carcinoma, lung adenocarcinoma, breast invasive carcinoma, ovarian serous cystadenocarcinoma, uterine corpus endometrioid carcinoma, esophageal carcinoma, liver hepatocellular carcinoma, bladder urothelial carcinoma, and sarcoma, at least one of the genes encoding the ufmylation factors (UBA5, UFC1, and UFL1) were amplified, while the gene encoding UfSP2, the only known deufmylation factor, sometimes was deleted, in more than 3% of patient cases (Table 1). However, despite the amplification of *UFC1* in pancreatic adenocarcinoma and amplification of both *UFC1* and *UFL1* in diffuse large B-cell lymphoma, high percentage of *UFL1* deletion and *UFL1*/*UFM1* deletion was also detected in pancreatic adenocarcinoma and diffuse large B-cell lymphoma, respectively (Table 1). It is of note that high percentage of *UFL1*/*UFM1* deletion was detected in prostate adenocarcinoma as well (Table 1). These molecular epidemiological datasets further support the observation that the ufmylation process may have opposite effects on tumorigenesis. Given that only one E3 for ufmylation has been identified so far, we believe that more E3 ligases await exploration.

## Conclusions

The UFM1 modification is a brand-new and very attractive Ub-like modification. With the very limited knowledge of ufmylation targets, we are far from appreciation of its molecular impact on cellular activities and etiology of human diseases, as well as its implication as therapeutic targets. We are very keen to understand (1) what the additional E3 ligases for ufmylation are; (2) what the additional ufmylation substrates are; (3) in addition to ER stress response and hematopoietic development, what other cellular activities the reversible ufmylation pathway regulates; (4) what the molecular mechanisms of the opposite effects on tumorigenesis are; (5) which ufmylation factor(s) is (are) an ideal biomarker and therapeutic target for certain types of cancer. Hence, the sleeping beauty of ufmylation is waiting for the awakening.

## Competing interests

The authors have declared no competing interests.

## Figures and Tables

**Table 1:** Abnormality of the ufmylation-related genes in different cancers

**Cancer type**	**No. of cases**	***UBA5***	***UFC1***	***UFL1***	***UfSP2***	***UFM1***
Lung squamous cell carcinoma	178	**11.8% (A)**	**7.3% (A)**	1.7% (M), 1.7% (D)	**5.6% (D)**	1.1% (D)
Lung adenocarcinoma	230	1.7% (A)	**12.6% (A)**	0.9% (M), 0.9% (D)	2.6% (D)	0.4% (A),1.3 (D)
Breast invasive carcinoma	974	0.9% (A)	**13.6% (A)**	1.1% (A)	1.8% (D)	1.7% (D), 0.3% (A)
Ovarian serous cystadenocarcinoma	311	**6.4% (A)**	**5.5% (A)**	1% (A)	**3.2% (D)**	1% (D), 0.6% (A), 0.3% (M)
Uterine corpus endometrioid carcinoma	240	1.3% (M)	**5% (A)**	**3.8% (M)**	**4.6% (M)**	0.4% (D)
Pancreatic adenocarcinoma	109	1.8% (D)	**21.1% (A)**	**7.3% (D)**	**4.6% (D)**	1.8% (D), 1.8% (A)
Esophageal carcinoma	92	**8.7% (A)**	**4.3% (A)**	1.1% (D)	2.2% (A)	1.1% (D)
Liver hepatocellular carcinoma	193		**15.5% (A)**	1.6% (D)	**3.6% (D)**	0.5% (D), 0.5% (A), 0.5% (M)
Bladder urothelial carcinoma	127	1.6% (A)	**18.9% (A)**	1.6% (M), 1.6% (D)	2.4% (M)	2.4% (D), 1.6% (A), 0.8% (M)
Sarcoma	256	0.4% (A)	**6.6% (A)**	1.2% (A)	**3.9% (D)**	1.6% (A), 1.6% (D)
Lymphoid neoplasm diffuse large B-cell lymphoma	48	**4.2% (A)**	**4.2% (A)**	**14.6% (D)**	**8.3% (D)**	**4.2% (D)**, 2.1% (A)
Prostate adenocarcinoma	420	1.7% (A)	1.2% (D)	**12.9% (D)**	0.2% (D)	**11.9% (D)**

*Note:* Data were obtained from www.cbioportal.org in December 2015. Alterations with frequency >3% are highlighted in bold. UFM1, ubiquitin-fold modifier 1; UfSP, UFM1-specific protease; UBA5, ubiquitin-like modifier-activating enzyme 5; UFC1, UFM1-conjugating enzyme 1; UFL1, UFM1-specific ligase 1; A, amplification; D, deletion; M, mutation.
